# Prolonged high-density culture with restricted medium renewal induces epithelial-like plasticity in apical papilla stem cells

**DOI:** 10.17305/bb.2026.14200

**Published:** 2026-05-11

**Authors:** Kameliya Kercheva, Marina Miteva, Silvia Kalenderova, Vanyo Mitev, Zornitsa Mihaylova, Nikolay Ishkitiev, Violeta S Dimitrova

**Affiliations:** 1 Department of Medical Chemistry and Biochemistry, Medical University-Sofia, Sofia, Bulgaria; 2 Research Institute of Innovative Medical Science, Medical University-Sofia, Sofia, Bulgaria; 3 Department of Dental, Oral and Maxillofacial Surgery, Medical University-Sofia, Sofia, Bulgaria

**Keywords:** Mesenchymal-epithelial transition, high-density culturing, stem cells from the apical papilla, RT^2^ Profiler PCR Array, microenvironmental stress

## Abstract

Stem cells from the apical papilla (SCAPs) are dental mesenchymal stem cells known for their high proliferative and multilineage potential. However, prolonged *in vitro* expansion may alter their phenotype due to microenvironmental stress. This exploratory study aimed to investigate whether prolonged high-density culture with restricted medium renewal is associated with morphological, protein-level, and transcriptional changes indicative of partial mesenchymal-epithelial transition (MET) in SCAPs. Donor-derived SCAPs were cultured under standard or prolonged high-density conditions. Morphological assessment was conducted using phase-contrast microscopy, protein expression was evaluated through immunofluorescence, and gene expression was analyzed using a Human epithelial-mesenchymal transition (EMT) reverse-transcription quantitative polymerase chain reaction (RT-qPCR) array. Prolonged culture resulted in a transition from spindle-shaped mesenchymal cells to compact epithelial-like clusters. This morphological change was accompanied by the upregulation of epithelial-associated genes, such as cadherin 1 (*CDH1*) and keratin 14 (*KRT14*), alongside the downregulation of extracellular matrix (ECM) and adhesion-related genes, including collagen type I alpha 2 chain (*COL1A2*), collagen type V alpha 2 chain (*COL5A2*), fibronectin 1 (*FN1*), and several integrins. Key regulators associated with EMT, including snail family transcriptional repressor 1 (*SNAI1*), snail family transcriptional repressor 3 (*SNAI3*), zinc finger E-box-binding homeobox 1 (*ZEB1*), notch receptor 1 (*NOTCH1*), and epidermal growth factor receptor (*EGFR*), were downregulated. In contrast, bone morphogenetic proteins 2 (*BMP2*) and 7 (*BMP7*), transforming growth factor beta 2 (*TGFB2*), matrix metalloproteinases 2 (*MMP2*) and 3 (*MMP3*), and secreted phosphoprotein 1 (*SPP1*) were upregulated. Immunofluorescence further demonstrated the expression of enamel-associated proteins, including amelogenin and ameloblastin, in long-term cultures. In conclusion, prolonged high-density culture with limited medium renewal was associated with coordinated epithelial-like phenotypic and transcriptional changes in SCAPs, suggesting the potential for partial mesenchymal-epithelial plasticity and emphasizing the sensitivity of dental stem cells to the culture microenvironment during *in vitro* expansion.

## Introduction

Dental mesenchymal stem cells (dMSCs) comprise a heterogeneous group of multipotent stromal cells, including dental pulp stem cells (DPSCs), periodontal ligament stem cells (PDLSCs), stem cells from human exfoliated deciduous teeth (SHED), and stem cells from the apical papilla (SCAPs). These cell populations exhibit significant abilities for proliferation, self-renewal, and multilineage differentiation, coupled with relatively low immunogenicity, positioning them as promising candidates for regenerative therapies [1, 2]. SCAPs represent a distinct subset of dMSCs located in the apical region of developing tooth roots [3, 4]. Typically isolated from immature permanent teeth, SCAPs display a mesenchymal-like morphology characterized by spindle-shaped cell bodies and elongated cytoplasmic processes [3, 5].

Epithelial–mesenchymal transition (EMT) and its counterpart, mesenchymal–epithelial transition (MET), are reversible biological processes that enable cells to switch between epithelial and mesenchymal phenotypes. EMT is characterized by the loss of epithelial polarity and cell–cell adhesion, accompanied by enhanced migratory and invasive capacities. Conversely, MET restores epithelial characteristics, including junctional integrity and proliferative potential. These transitions play fundamental roles in embryogenesis, tissue repair, and organ regeneration, and they are also implicated in pathological processes such as fibrosis and cancer metastasis [6, 7].

EMT can be classified into three subtypes: type I EMT occurs during embryonic development and organogenesis; type II EMT is involved in wound healing, tissue repair, and fibrosis; and type III EMT is associated with tumor progression and metastasis [7]. MET, as the reverse process, allows mesenchymal cells to regain epithelial traits and is increasingly recognized as a crucial mechanism underlying tissue regeneration and cellular plasticity [6].

At the molecular level, EMT is characterized by the downregulation of epithelial markers such as E-cadherin and cytokeratins, and the induction of mesenchymal genes including fibronectin and N-cadherin. The expression of vimentin may vary based on cellular context [8, 9]. Conversely, MET involves the reactivation of epithelial gene programs, specifically the upregulation of E-cadherin (CDH1) and cytokeratins, alongside the suppression of mesenchymal markers such as N-cadherin (CDH2) and vimentin (VIM), resulting in the restoration of epithelial polarity and junctional organization [8]. These transitions are regulated by EMT-inducing transcription factors, including snail family transcriptional repressor 1 (SNAI1)/snail family transcriptional repressor 2 (SNAI2), zinc finger E-box-binding homeobox 1 (ZEB1)/zinc finger E-box-binding homeobox 2 (ZEB2), and TWIST, whose repression facilitates epithelial gene expression. The miR-200/ZEB pathway is a key regulatory axis promoting MET, where miR-200 family members inhibit ZEB1/2 and stabilize epithelial identity. Furthermore, signaling molecules that counteract EMT, such as bone morphogenetic protein 7 (BMP7), have been shown to induce MET-like changes in various biological systems [10, 11].

Emerging evidence suggests that microenvironmental conditions can significantly influence the fate decisions of mesenchymal stem cells. Factors such as mechanical stress, availability of oxygen and glucose, and matrix composition impact cytoskeletal organization, transcriptional programs, and lineage commitment [12, 13]. In SCAPs, adverse culture conditions, such as nutrient and oxygen deprivation, induce notable phenotypic plasticity, including endothelial-like shifts and activation of angiogenic programs, underscoring the adaptability of this cell population [3]. Additionally, conventional two-dimensional culture systems fail to replicate the complexity of the native tissue niche, contributing to cellular heterogeneity and a gradual loss of stemness during expansion [14].

While EMT and MET have been extensively characterized in developmental and pathological contexts, the effects of prolonged *ex vivo* culture stress on EMT/MET-related plasticity in dMSCs, particularly SCAPs, remain poorly understood. Specifically, it is unclear whether extended high-density culture conditions induce coordinated transcriptional and phenotypic changes consistent with a partial MET-like phenotype, or how these changes may influence SCAP identity and behavior.

The aim of this study is to determine whether prolonged high-density culture under conditions of limited medium exchange is associated with morphological alterations and transcriptional changes indicative of a partial MET in SCAPs. We evaluate the effects of extended culture on cellular morphology and the expression of selected epithelial and mesenchymal markers.

## Materials and methods

### Isolation and cultivation of SCAPs

SCAPs were isolated and cultivated following established protocols [15]. Cells were sourced from the apical papilla of individuals under 25 years of age, with no systemic diseases and healthy oral status. Inclusion criteria required the presence of intact teeth without inflammation or carious lesions, irrespective of eruption status. These teeth are typically extracted as part of orthodontic treatment and may contribute to complications in occlusal development. Exclusion criteria encompassed teeth with cavities or fillings, various inflammatory conditions such as pulpitis and periodontitis (acute and chronic), and periapical infections, leading to the exclusion of the respective teeth from the study [15].

SCAP cultures were established using previously described protocols routinely employed in our laboratory for the isolation and characterization of dental stem cells. The identity of SCAPs was confirmed through immunofluorescence staining for established markers, including CD24, STRO-1, and CD146, as outlined in prior studies [16]. Additionally, the cells exhibited a characteristic fibroblast-like morphology consistent with SCAP populations. Representative validation data are available in our previous publication [16].

For this experiment, SCAP cultures were established from three independent donors. Second-passage SCAPs for each donor were maintained under standard conditions (37 ^∘^C, 5% CO_2_) and assigned to two different culture conditions (paired design): control SCAPs and experimental SCAPs, which differed significantly in culture regimen and duration. Control SCAPs were maintained under standard conditions with routine passaging at 60%–70% confluence and complete medium renewal every three days. In contrast, experimental SCAPs were cultured at high density (3 × 10^4^ cells/cm^2^) and maintained at sustained confluence in 75 cm^2^ culture flasks with restricted medium renewal (every ten days) and extended intervals between passages (3–4 weeks). The total culture duration for SCAPs in experimental conditions, from passage 1 (P1) to passage 7 (P7), was approximately 21 weeks, compared to approximately 9 weeks for control SCAP cultures maintained under standard conditions. Cellular morphology was assessed by phase-contrast microscopy at each passage (Leica DMRE, Leica Microsystems GmbH).

This design was intentionally employed to model cumulative microenvironmental stress associated with long-term culturing, including high cell density, prolonged confluence, and reduced medium renewal. Consequently, the two conditions differ not only in cell density but also in cumulative culture duration and exposure to microenvironmental stress, warranting interpretation as a combined stress model rather than the impact of a single isolated variable.

### Ethical statement

The study was approved by the Ethics Committee of Medical University–Sofia, Bulgaria. All patients (or their guardians) participating in the study signed an informed consent form prior to sample extraction, in accordance with the decision of the Ethical Committee of Medical University Sofia’s Council of Medical Science (No. 3573∖06.08.2024). All tooth extractions were performed at the Department of Dental, Oral, and Maxillofacial Surgery within the Faculty of Dental Medicine, Medical University Sofia, following thorough orthodontic and surgical consultations. No teeth were extracted solely for this study.

### RT profiler PCR array

Total RNA was isolated from control SCAPs and experimental SCAPs cultured in 75 cm^2^ flasks at passage 7 using the RNeasy Mini Kit (QIAGEN, USA), following the manufacturer’s instructions. Complementary DNA (cDNA) was synthesized using the RT^2^ First Strand Kit (QIAGEN). RNA was isolated separately from each donor-derived culture; however, equal amounts of RNA from the three donors were pooled prior to analysis.

Gene expression profiling was conducted using the QIAGEN Human EMT RT^2^ Profiler PCR Array (Cat. No. PAHS-090Z), which quantifies the expression of 84 genes associated with epithelial–mesenchymal and mesenchymal–epithelial phenotypes. Real-time PCR was performed in accordance with the manufacturer’s protocol. The array encompasses mesenchymal-associated markers (e.g., VIM, CDH2, collagens), epithelial-associated markers (e.g., CDH1 and cytokeratins), EMT-related transcription factors (e.g., SNAI1, SNAI2, twist family bHLH transcription factor 1 [TWIST1], ZEB1, ZEB2), and signaling molecules involved in critical pathways, including transforming growth factor beta (TGF-β), Wnt, and Notch. A complete list of genes included in the EMT RT^2^ Profiler PCR Array is provided in Supplemental data 1.

### Statistical analysis

Cycle threshold (Ct) values from the RT Profiler Array were uploaded and analyzed using the QIAGEN GeneGlobe Data Analysis Center. Candidate housekeeping genes, including actin beta (*ACTB*), beta-2-microglobulin (*B2M*), glyceraldehyde-3-phosphate dehydrogenase (*GAPDH*), ribosomal protein lateral stalk subunit P0 (*RPLP0*), and hypoxanthine phosphoribosyltransferase 1 (*HPRT1*), were assessed for expression stability utilizing RefFinder, an online platform that integrates geNorm, NormFinder, BestKeeper, and the comparative delta cycle threshold (ΔCt) method [17]. The selection of reference genes was grounded in overall consistency across these methods rather than a singular criterion.

The ΔCt method identified RPLP0, B2M, and GAPDH as the most stable genes (average SD = 0.90), while HPRT1 exhibited significant variability (SD = 2.41). BestKeeper analysis revealed that B2M had the lowest variability (SD = 0.90), whereas GAPDH (SD = 1.11) and RPLP0 (SD = 1.28) displayed moderately greater variability but maintained a strong correlation with the BestKeeper index (r ≥ 0.988, *P* ≤ 0.001). NormFinder ranked GAPDH (0.200) and B2M (0.215) as the most stable genes, followed by RPLP0 (0.439). Similarly, geNorm identified RPLP0 and ACTB as the most stable pair (*M* ═ 0.241); however, the inconsistent performance of ACTB across methods precluded its selection. Overall, B2M and GAPDH demonstrated consistent high stability across methods, while RPLP0 exhibited comparable performance. In contrast, ACTB and HPRT1 were characterized by low or inconsistent stability and were subsequently excluded. Thus, B2M, GAPDH, and RPLP0 were selected for normalization.

Gene expression changes were calculated using the ΔΔCt method, comparing control and experimental SCAP cultures (Supplemental data 1). Results are presented as fold changes, with values greater than 1 indicating upregulation and values below 1 indicating downregulation. Since RNA samples were pooled prior to analysis, statistical testing at the gene level was not conducted. Consequently, the results are considered exploratory and should be interpreted based on fold changes and overall expression patterns.

For clarity, genes were categorized into functional groups, including mesenchymal markers, epithelial markers, EMT/MET-associated transcription factors, and signaling molecules.

### Pathway analysis with reactome

Pathway enrichment analysis was conducted using the Reactome Pathway Database (https://reactome.org) as an exploratory method to provide pathway-level context for the observed gene expression patterns. Genes exhibiting differential expression based on fold-change criteria identified from the RT^2^ Profiler PCR Array were uploaded into the Reactome Analysis Tool. Enrichment results were reported as raw *P*-values and false discovery rate (FDR)–adjusted *P*-values, with pathways having FDR ≤ 0.05 deemed enriched (Supplemental data 2).

### Immunofluorescence

For immunofluorescence staining, cells were plated in 6- or 12-well plates (TPP, Switzerland) at a density of 5,000 cells/cm^2^. Upon reaching subconfluence, cells were fixed in 4% formaldehyde for 30 min, rinsed three times with phosphate-buffered saline (PBS; Thermo Fisher Scientific, USA), and subsequently blocked with 1% bovine serum albumin (BSA) in PBS for an additional 30 min. To facilitate intracellular antigen staining, cells were permeabilized with 0.05% Tween-20 (ICN Biomedicals Inc., UK) for 10 min, followed by 0.05% Triton X-100 (Merck, Germany) for 30 min. Cells were then indirectly immunofluorescently labeled using primary antibodies: mouse monoclonal antibodies against vimentin (sc-58899; 1:100, Santa Cruz Biotechnology, USA), ameloblastin (sc-271012; 1:100, Santa Cruz), and amelogenin (sc-365284; 1:100, Santa Cruz), as well as rabbit antibodies against cytokeratin 14 (CK14) (AN300733L; 1:100, Elabscience, USA), cytokeratin 19 (CK19) (ab76539; 1:100, Abcam, UK), and collagen type III (COL3A1; 1:100, SAB4500367; Merck, Germany) [18, 19]. Following this, incubation with secondary antibodies occurred: anti-mouse IgG conjugated with AlexaFluor 488 (A32766; 1:1000, Thermo Fisher Scientific, USA) and anti-rabbit IgG conjugated with CruzFluor 555 (sc362272; 1:1000, Santa Cruz) overnight at 4 ^∘^C, followed by three washes with PBS. All antibodies were utilized at working concentrations as per the manufacturers’ instructions. Nuclei were counterstained with 0.1 µg/mL of 4′,6-diamidino-2-phenylindole (DAPI; Invitrogen, USA) for 15 min and subsequently washed three times with PBS. Images were captured using an INCell Analyzer 6000 imaging system with a laser confocal slit mechanism (GE Healthcare, USA).

## Results

### Phase-contrast microscopy

**Figure 1.**
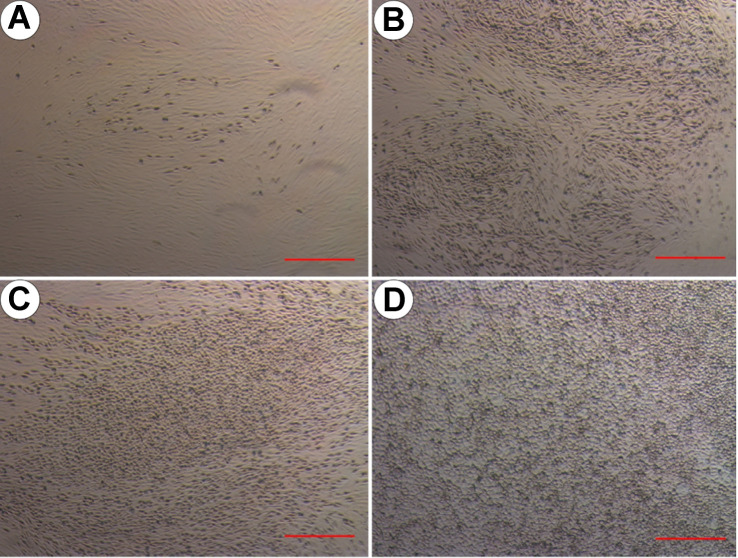
**Progressive epithelial-like morphological shift in experimental SCAP cultures during prolonged high-density culturing with restricted medium renewal.** Representative phase-contrast microscopy images show experimental SCAP cultures at passage 4 (A), passage 5 (B), passage 6 (C), and passage 7 (D). At passage 4, cells predominantly retained an elongated, spindle-shaped mesenchymal-like morphology. With continued culture, SCAPs progressively became more densely arranged and displayed a rounded, compact, cobblestone-like appearance, consistent with an epithelial-like morphological shift. By passage 7, most cells showed a dense epithelial-like growth pattern. Scale bars = 500 µm. Abbreviation: SCAPs, stem cells from the apical papilla.

Experimental group SCAPs from all three donors exhibited progressive alterations in cell morphology during prolonged high-density culture and limited medium renewal. Cells transitioned from an elongated, spindle-shaped mesenchymal morphology to a more rounded, cobblestone-like epithelial appearance, with initial changes becoming evident at passage 4 and progressively increasing in subsequent passages. By passage 7, the majority of cells displayed this epithelial-like morphology. These morphological changes were consistently observed across all biological replicates and multiple microscopic fields. Following passage, cells maintained an epithelial-like morphology under long-term culture conditions (Figure 1). In contrast, serial passaging under control conditions did not reveal detectable changes in cell morphology, and control SCAPs preserved a stable fibroblast-like phenotype throughout the experimental period.

### Global gene expression changes

Experimental SCAPs exhibited a distinct transcriptional profile compared to the control group. The observed gene expression patterns were consistent with down-regulation of canonical mesenchymal programs alongside increased expression of several epithelial-associated transcripts, accompanied by alterations related to cytoskeletal remodeling and modified cell–matrix interactions.

**Figure 2.**
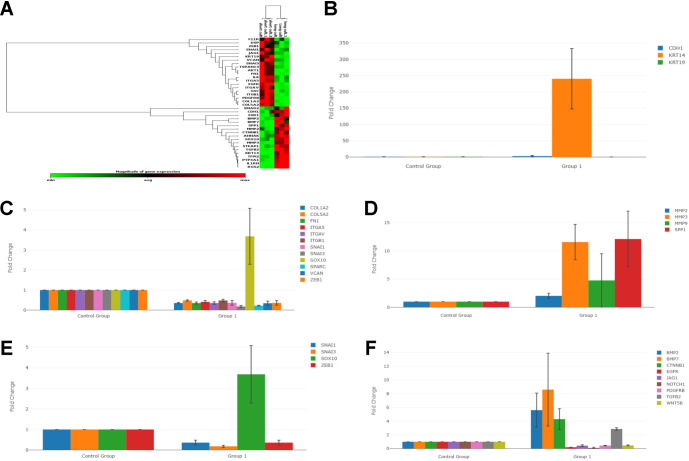
**Transcriptional reprogramming of SCAPs under prolonged high-density culture with restricted medium renewal.** Gene expression profiling was performed using the Human EMT RT^2^ Profiler PCR Array and analyzed with the QIAGEN GeneGlobe Data Analysis Center. (A) Hierarchical clustergram showing distinct separation between control SCAPs and experimental SCAPs based on EMT/MET-associated gene expression patterns. (B) Epithelial-associated markers showed increased expression of *CDH1* and a marked increase in *KRT14*, whereas KRT19 was decreased in experimental SCAPs. (C) Mesenchymal, ECM, and adhesion-related genes showed an overall reduction in experimental SCAPs, including *COL1A2, COL5A2, FN1, ITGA5, ITGAV, ITGB1, VCAN*, and *SPARC*. (D) Matrix-remodeling and stress-associated genes showed increased expression, particularly *MMP3, MMP9*, and *SPP1*. (E) EMT-related transcription factors showed reduced expression of *SNAI1, SNAI3*, and *ZEB1*, whereas SOX10 was increased. (F) Signal transduction-related genes showed increased expression of *BMP2, BMP7, CTNNB1*, and *TGFB2*, with reduced expression of *EGFR, JAG1, NOTCH1, PDGFRB*, and *WNT5B*. Fold changes are shown relative to control SCAPs. Data are based on pooled samples and are presented descriptively within this exploratory study; therefore, gene-level statistical testing was not performed. Control group, control SCAPs; Group 1, experimental SCAPs. Abbreviations: BMP2, bone morphogenetic protein 2; BMP7, bone morphogenetic protein 7; CDH1, cadherin 1; COL1A2, collagen type I alpha 2 chain; COL5A2, collagen type V alpha 2 chain; CTNNB1, catenin beta 1; ECM, extracellular matrix; EGFR, epidermal growth factor receptor; EMT, epithelial–mesenchymal transition; FN1, fibronectin 1; ITGA5, integrin subunit alpha 5; ITGAV, integrin subunit alpha V; ITGB1, integrin subunit beta 1; JAG1, jagged canonical Notch ligand 1; KRT14, keratin 14; KRT19, keratin 19; MET, mesenchymal–epithelial transition; MMP3, matrix metalloproteinase 3; MMP9, matrix metalloproteinase 9; NOTCH1, notch receptor 1; PDGFRB, platelet-derived growth factor receptor beta; SCAPs, stem cells from the apical papilla; SNAI1, snail family transcriptional repressor 1; SNAI3, snail family transcriptional repressor 3; SOX10, SRY-box transcription factor 10; SPARC, secreted protein acidic and cysteine rich; SPP1, secreted phosphoprotein 1; TGFB2, transforming growth factor beta 2; VCAN, versican; WNT5B, Wnt family member 5B; ZEB1, zinc finger E-box binding homeobox 1.

Hierarchical clustering (Figure 2A) demonstrated a clear separation between control and experimental conditioned SCAPs. Clustering analysis was performed on technical replicates of pooled samples and is presented for descriptive visualization within this exploratory study.

### Epithelial markers

In the experimental SCAP culture, CDH1 demonstrated increased expression (fold change 2.8), alongside a significant rise in the basal epithelial keratin 14 (KRT14) (fold change 240; ΔCt 17.08 vs. 9.17). In contrast, the expression of luminal epithelial keratin 19 (*KRT19*) decreased (0.12) (Figure 2B).

### Mesenchymal markers and extracellular matrix (ECM)/adhesion genes

Genes associated with ECM organization and mesenchymal cell–matrix interactions exhibited reduced expression in experimental SCAPs. Several integrins involved in cell–matrix adhesion, including integrin subunit alpha 5 (*ITGA5*) (0.41), integrin subunit alpha V (*ITGAV*) (0.35), and integrin subunit beta 1 (*ITGB1*) (0.48), displayed lower expression compared to control SCAPs. Additionally, key ECM structural and matricellular transcripts were decreased, including collagen type I alpha 2 chain (*COL1A2*) (0.35), collagen type V alpha 2 chain (*COL5A2*) (0.48), fibronectin 1 (*FN1*) (0.35), versican (*VCAN*) (0.34), and secreted protein acidic and cysteine rich (*SPARC*) (0.22) (Figure 2C).

**Table 1:** Enriched reactome pathways in experimental SCAPs

**Pathway name**	**Entities**				**Representative genes**
	**found**	**ratio**	*P* **value**	FDR*	
ECM proteoglycans	8/79	0.005	1.49e-08	6.27e-07	*COL1A2, COL5A2, FN1, ITGAV, ITGB1,SPARC,TGFB2,VCAN*
Fibronectin matrix formation	5 / 21	0.001	1.33e-07	4.08e-06	*FN1, ITGA5, ITGB1, COL1A2, COL5A2*
Degradation of the extracellular matrix	9 / 148	0.009	1.36e-07	4.08e-06	*BMP1, COL5A2, COL1A2, MMP3, MMP9, CDH1, FN1, SPP1*
Interleukin-4 and interleukin-13 signaling	16 / 211	0.013	4.31e-14	5.51e-12	*AKT1, FN1, ITGB1, COL1A2, IL1RN, MMP2, MMP3, ZEB1*
Developmental cell lineages	18 / 283	0.018	1.83e-14	4.69e-12	*CDH1, EGFR, KRT14, KRT19, ESR1, GSC, TFPI2*
Developmental lineage of mammary gland myoepithelial cells	8 / 35	0.002	2.69e-11	1.96e-09	*CDH1, ITGB1, EGFR, KRT14*
Developmental lineage of pancreatic ductal cells	7 / 77	0.005	2.54e-07	6.16e-06	*KRT14, KRT19, TFPI2, FN1, COL5A2, COL1A2, TFPI2*
Formation of definitive endoderm	5 / 25	0.002	3.13e-07	7.21e-06	*CDH1, CTNNB1, GSC*

To enhance biological interpretability, pathways were categorized by functional groups while preserving FDR-based ranking within each category. Pathway enrichment analysis was conducted using Reactome, focusing on genes selected based on fold-change criteria from pooled samples. Statistical values, including *P* values and FDR, were generated by the Reactome analysis tool and applied at the pathway level.Representative genes contributing to each pathway are provided. The results are presented for exploratory interpretation. Abbreviations: AKT1, AKT serine/threonine kinase 1; BMP1, bone morphogenetic protein 1; CDH1, cadherin 1; COL1A2, collagen type I alpha 2 chain; COL5A2, collagen type V alpha 2 chain; CTNNB1, catenin beta 1; ECM, extracellular matrix; EGFR, epidermal growth factor receptor; ESR1, estrogen receptor 1; FDR, false discovery rate; FN1, fibronectin 1; GSC, goosecoid homeobox; IL1RN, interleukin 1 receptor antagonist; ITGA5, integrin subunit alpha 5; ITGAV, integrin subunit alpha V; ITGB1, integrin subunit beta 1; KRT14, keratin 14; KRT19, keratin 19; MMP2, matrix metalloproteinase 2; MMP3, matrix metalloproteinase 3; MMP9, matrix metalloproteinase 9; SCAPs, stem cells from the apical papilla; SPARC, secreted protein acidic and cysteine rich; SPP1, secreted phosphoprotein 1; TGFB2, transforming growth factor beta 2; TFPI2, tissue factor pathway inhibitor 2; VCAN, versican; ZEB1, zinc finger E-box binding homeobox 1.

**Figure 3.**
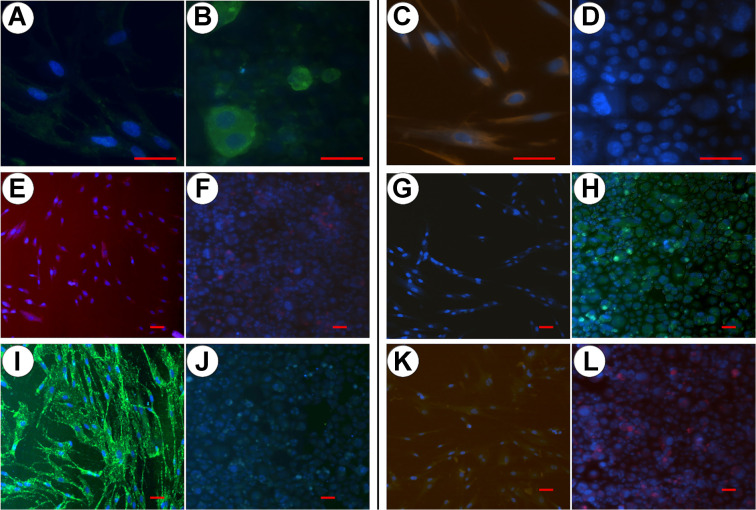
**Immunofluorescence characterization of control and experimental SCAP cultures.** Representative immunofluorescence images comparing control SCAPs (A, C, E, G, I, K) and experimental SCAPs (B, D, F, H, J, L) after prolonged high-density culturing with restricted medium renewal. CK14 staining (green) is shown in control (A) and experimental (B) SCAPs at ×60 magnification and demonstrates stronger expression in experimental SCAPs. Vimentin staining (orange) is shown in control (C) and experimental (D) SCAPs at ×60 magnification and appears reduced in experimental SCAPs. CK19 staining (red) is shown in control (E) and experimental (F) SCAPs at ×20 magnification and is decreased in experimental SCAPs. Amelogenin staining (green) is shown in control (G) and experimental (H) SCAPs at ×20 magnification and is detectable in experimental SCAPs. *COL3A1* staining (green) is shown in control (I) and experimental (J) SCAPs at ×20 magnification and appears reduced in experimental SCAPs. Ameloblastin staining (red) is shown in control (K) and experimental (L) SCAPs at ×20 magnification and is detectable in experimental SCAPs. Nuclei were counterstained with DAPI (blue). Scale bars = 20 µm. Abbreviations: CK14, cytokeratin 14; CK19, cytokeratin 19; COL3A1, collagen type III alpha 1 chain; DAPI, 4′,6-diamidino-2-phenylindole; SCAPs, stem cells from the apical papilla.

### Stress-associated and matrix-remodeling response

In conjunction with decreased expression of ECM and adhesion-related genes, experimental SCAPs exhibited increased expression of several transcripts associated with cellular stress and matrix remodeling. Notably, AHNAK nucleoprotein (*AHNAK*) (fold change ∼100; ΔCt 8.48 in experimental SCAPs vs. 1.84 in control SCAPs) and secreted phosphoprotein 1 (SPP1) (fold change 12.13; ΔCt 13.57 vs. 9.97) were markedly upregulated. Furthermore, elevated expression of matrix metalloproteinases *MMP2* (2.03), *MMP3* (fold change 11.59; ΔCt 10.65 vs. 7.11), and *MMP9* (4.77) was observed (Figure 2D).

### EMT-related transcription factors

Experimental SCAPs displayed reduced expression of several transcription factors associated with epithelial–mesenchymal transition, including SNAI1, snail family transcriptional repressor 3 (SNAI3), and ZEB1. In contrast, SRY-box transcription factor 10 (*SOX10*) exhibited increased expression and was the only transcriptional regulator to show this pattern (3.69) (Figure 2E).

### Signal transduction molecules

Genes implicated in EMT/MET-associated signaling exhibited distinct expression changes under high-density culture conditions. The components of the TGF/bone morphogenetic protein (BMP) axis exhibited increased expression levels, specifically transforming growth factor beta 2 (TGFB2) (2.87), bone morphogenetic protein 2 (BMP2) (5.61), and BMP7 (8.60). Conversely, several growth factor– and Notch-related genes showed decreased expression, including epidermal growth factor receptor (*EGFR*) (0.20), notch receptor 1 (*NOTCH1*) (0.11), platelet-derived growth factor receptor beta (*PDGFRB*) (0.45), jagged canonical Notch ligand 1 (*JAG1*) (0.44), and Wnt family member 5B (*WNT5B*) (0.47). Additional signaling regulators also exhibited altered expression, including catenin beta 1 (*CTNNB1*) (4.30), regulator of G protein signaling 2 (*RGS2*) (9.99), and tissue factor pathway inhibitor 2 (*TFPI2*) (3.41) (Figure 2F).

### Reactome pathway enrichment analysis

Reactome pathway analysis was employed as an exploratory tool to elucidate the biological context of the observed gene expression patterns. Notable pathways related to the ECM, such as ECM proteoglycans and fibronectin matrix formation, were highlighted, featuring genes like FN1, ITGB1, ITGAV, COL1A2, and COL5A2. Additionally, developmental pathways pertinent to mammary gland and integumentary system development were represented. Cytokine-related pathways, including interleukin-4 and interleukin-13 signaling, were also identified, encompassing genes involved in signaling and matrix remodeling, such as AKT serine/threonine kinase 1 (AKT1), FN1, ITGB1, COL1A2, MMP2, MMP3, ZEB1, and interleukin 1 receptor antagonist (IL1RN). These results are exploratory in nature and offer pathway-level context for the observed gene expression patterns (Table 1).

### Immunofluorescence

In experimental SCAPs, a significant alteration in cell morphology was noted, with cells forming more compact, epithelial-like clusters. Immunofluorescence analysis demonstrated increased expression of the epithelial marker CK14 (Figure 3B) and decreased expression of CK19 (Figure 3F) in experimental SCAPs (Figure 3A, 3E). The expression of the mesenchymal marker vimentin (Figure 3D) and the ECM-associated marker COL3A1 (Figure 3J) was reduced compared to control SCAPs (Figure 3C, 3I).

Moreover, experimental SCAPs exhibited detectable expression of enamel-related proteins amelogenin (Figure 3H) and ameloblastin (Figure 3L), which were minimally expressed or absent in control SCAP cultures (Figure 3G, 3K).

Collectively, these observations indicate that experimental SCAPs display altered protein markers and gene expression patterns relative to control SCAP cultures.

## Discussion

This study investigated the effects of prolonged high-density culture with limited medium renewal on the morphology and gene expression profiles of SCAPs. Our findings reveal that extended culture under these conditions is associated with morphological, immunocytochemical (protein-level), and transcriptional changes in epithelial- and mesenchymal-associated genes, indicative of a partial epithelial-like shift. To our knowledge, similar findings have not been previously reported in SCAP cultures. These changes occurred without exogenous differentiation cues or chemical stressors, suggesting that cumulative culture-associated factors may significantly influence SCAP phenotype during long-term *in vitro* expansion. Prolonged confluent culture was associated with transcriptional changes consistent with mesenchymal–epithelial plasticity, including elements of an MET-like transcription program in SCAPs, thereby supporting the notion that cellular microenvironmental conditions can influence stem cell phenotype *in vitro*.

### Changes in mesenchymal and epithelial related marker expression

RT^2^ Profiler analysis demonstrated an increase in *CDH1* expression, which encodes E-cadherin, a crucial adhesion molecule mediating adherens junctions in epithelial cells. Elevated *CDH1* expression is associated with the acquisition or stabilization of an epithelial phenotype during mesenchymal-to-epithelial transitions [20]. In a human embryonic stem cell differentiation model to hepatocytes, *CDH1* expression exhibited an inverse relationship with mesenchymal markers such as CDH2, VIM, and SNAI1, decreasing when these markers were upregulated during intermediate EMT-like stages and increasing again upon the acquisition of a mature epithelial-like phenotype [21]. Similarly, studies on pluripotent stem cells indicate that downregulation of *CDH1* is a key initiating event of EMT, whereas its maintenance or re-expression is linked to epithelial stabilization and an MET-like phenotype [22, 23].

In accordance with these observations, RT^2^ Profiler analysis also revealed changes in keratin gene expression consistent with epithelial stabilization. *KRT14*, a cytokeratin characteristic of stratified and odontogenic epithelia [24, 25], exhibited a marked increase in expression in experimental SCAPs (fold change ∼240), supported by a substantial difference in Ct values (17.08 vs. 9.17), while *KRT19* was reduced and keratin 7 (*KRT7*) remained unchanged. This pattern suggests epithelial reinforcement rather than differentiation toward a simple epithelial phenotype.

Protein-level analysis further corroborated these findings. Immunocytochemistry showed stronger CK14 staining in experimental SCAP cultures, consistent with increased *KRT14* transcription, while CK19 protein was undetected, aligning with reduced *KRT19* transcript levels.

Analysis of mesenchymal markers revealed additional changes at the protein level. Vimentin, a canonical intermediate filament associated with mesenchymal identity [26], was strongly positive in control SCAP cultures but exhibited reduced and more heterogeneous expression in experimental SCAPs. Together, the increased epithelial markers (*CDH1*, CK14) and diminished vimentin staining indicate a shift toward epithelial-like characteristics and may reflect aspects of mesenchymal–epithelial plasticity.

### Potential signaling mechanisms underlying MET-like changes in long-term culture

#### Suppression of EMT regulatory networks in experimental SCAP cultures

The transcriptional profile observed in experimental SCAP cultures suggests diminished activity of canonical EMT regulators. Expression of *NOTCH1* was reduced, accompanied by decreased expression of its downstream effectors *SNAI1*, *SNAI3*, and *ZEB1*. These transcription factors are well-established drivers of EMT, primarily functioning through the transcriptional repression of epithelial junctional genes such as *CDH1* (E-cadherin) [27–29]. *SNAI1* (Snail) is a key EMT transcription factor that directly suppresses E-cadherin expression, promoting the loss of epithelial cell adhesion and the acquisition of a mesenchymal phenotype [27, 30].

Consistent with this regulatory framework, studies have shown that loss of ZEB1 activity can facilitate MET, enabling re-expression of epithelial markers such as E-cadherin while concurrently reducing mesenchymal markers, including vimentin. ZEB1 functions as a transcriptional repressor of epithelial genes, and its loss results in the re-expression of epithelial markers and a reduction in mesenchymal features [31].

The observed reduction in *NOTCH1* expression may further contribute to this phenomenon. Notch signaling is known to promote EMT by activating transcription factors such as *SNAI1* and *SNAI2* (Snail and Slug), which repress epithelial adhesion molecules and enhance migratory behavior. Inhibition of Notch signaling has been associated with the reversal of EMT phenotypes and the promotion of MET-associated gene expression patterns, including increased E-cadherin and decreased vimentin or N-cadherin [32]. Additionally, interactions between ZEB1 and the miR-200 regulatory loop have been shown to modulate Notch signaling and EMT dynamics across various cellular systems [33].

In this context, the observed reduction in *NOTCH1*, *SNAI1/3*, and *ZEB1* expression in experimental SCAPs may indicate diminished EMT-associated signaling networks. Reduced activity of these transcriptional repressors is expected to alleviate the inhibition of epithelial gene programs, potentially enabling the re-expression of adhesion molecules such as E-cadherin and fostering epithelial-like cellular characteristics. NOTCH receptors (including *NOTCH3*) and their ligand JAG1 are normally expressed in SCAPs. The proportion of NOTCH3-positive cells declines during osteogenic differentiation, supporting a role for Notch signaling in maintaining a mesenchymal, progenitor-like state [34]. The observed changes align with features of partial MET-like transcriptional reprogramming rather than a complete lineage conversion.

#### Autocrine TGF-Δ activation

One potential explanation for the observed epithelial-like changes is the involvement of autocrine signaling mechanisms. Sustained high cell density may promote the accumulation and local release of TGF-β family ligands [35, 36]. In our dataset, increased expression of TGFB2 was noted, which encodes transforming growth factor-β2, a member of the TGF-β superfamily known to regulate epithelial–mesenchymal plasticity. Although TGF-β signaling is classically associated with EMT induction [37], its biological effects are context-dependent and necessitate the activation of latent ligands within the ECM [38]. Interestingly, expression of integrins commonly involved in canonical TGF-β activation [38], including *ITGA5*, *ITGAV*, and *ITGB1*, was reduced in our dataset. Concurrently, increased expression of CDH1 (E-cadherin) is expected to strengthen adherens junctions, while the concurrent downregulation of *ITGB1* and *ITGA5* likely diminishes integrin-mediated attachment to the ECM and attenuates pro-migratory signaling pathways [39].

*ITGA5*, *ITGAV*, and *ITGB1* have been identified as the most highly expressed integrins in SCAP [40]. Integrin α5β1 serves as a principal receptor for fibronectin, and its loss is associated with decreased FN1 expression and diminished focal adhesion kinase (FAK) activity [41]. This reduction may lead to decreased downstream activation of the mitogen-activated protein kinase (MAPK)/extracellular signal-regulated kinase (ERK) pathway, which could further inhibit ECM production. As a result, the expression of matrix protein genes, such as COL1A2 (a collagen component of a rigid niche) and matricellular factors VCAN and SPARC, is diminished, all of which support fibroblastic behavior and EMT [42].

In contrast to other TGF-β isoforms, TGF-β2 lacks the arginine-glycine-aspartic acid (RGD) motif necessary for integrin-mediated activation [43], indicating that alternative activation mechanisms may be relevant in this context. Protease-mediated activation of latent TGF-β has been reported, with matrix metalloproteinases (MMPs) such as *MMP2* and *MMP9* implicated in this process. Among TGF-β isoforms, TGF-β2 exhibits particular sensitivity to MMP9-mediated activation [44]. Our dataset shows increased expression of matrix-remodeling enzymes, suggesting that protease-dependent activation of latent TGF-β may contribute to signaling outcomes under prolonged culture conditions.

#### Metabolic stress and lactate-dependent activation

Another potential contributor to TGF-β activation during extended culture is the accumulation of lactate under high-density conditions. Limited medium exchange in high-density cultures may lead to lactate accumulation, a primary byproduct of glycolytic metabolism, resulting in progressive acidification once buffering capacity is exceeded. Lactate-induced acidification has been shown to activate latent TGF-β in a pH-dependent manner at physiologically relevant concentrations within fibrotic systems [45].

#### Density-dependent signaling and cell cycle attenuation in experimental SCAPs

Prolonged confluence and restricted medium exchange impose metabolic and spatial constraints that can promote contact inhibition and reduced cell cycle activity, as observed in long-term mesenchymal stem cell cultures [46]. Several transcriptional changes noted in our dataset align with diminished proliferative signaling during extended culture. For instance, the downregulation of EGFR and its downstream effector AKT1—key regulators of proliferative and EMT-associated signaling pathways—may reflect attenuation of mitogenic signaling under these conditions. This reduction in signaling activity may contribute to a transcriptional shift from mesenchymal signaling toward epithelial-like characteristics [47].

Density-dependent growth arrest is partially mediated by cell–cell adhesion and Hippo signaling pathways, which regulate proliferation in response to junctional architecture. The observed increase in *CDH1* expression may further reinforce contact inhibition through adherens junction formation and Hippo pathway signaling, both known to suppress proliferation [48].

#### BMP signaling as an EMT antagonist

Despite conditions that may promote TGF-β activation and EMT, our experiments revealed increased expression of *BMP7*, a well-established antagonist of TGF-β–driven EMT and a promoter of MET programs, suggesting that BMP-mediated signaling may play a dominant role. This antagonism is critical, as BMP-7 has been shown to inhibit TGF-β–induced myofibroblast transdifferentiation and to promote epithelial stabilization by supporting the membranous localization of epithelial markers such as E-cadherin and β-catenin. Furthermore, *BMP-7* overexpression attenuates transforming growth factor beta 1 (TGF-β1)–induced EMT through suppression of Wnt3/β-catenin and TGF-β/Smad2/3 signaling, highlighting a key mechanism by which BMP signaling can functionally counterbalance EMT programs [49, 50].

### ECM remodeling and reduced collagenous matrix deposition

The reduced expression of *COL1A2* and *COL5A2*, alongside decreased *FN1* expression, suggests diminished deposition of collagen-rich ECM in long-term SCAP cultures. This pattern parallels the increased expression of MMP2, MMP3, and MMP9, indicating active ECM remodeling under prolonged dense culture conditions. Our previous study identified collagen type I alpha 1 chain (*COL1A1*) and collagen type V alpha 1 chain (*COL5A1*) as the most expressed collagens in all dental mesenchymal stem cell types, including SCAPs, with MMP2 and MMP14 as the most expressed MMPs, followed by MMP3 and MMP9 in fifth and sixth positions, respectively [40].

Consistent with these transcriptional changes, immunocytochemical analysis revealed reduced protein levels of COL3A1 in experimental SCAPs, supporting alterations in collagen matrix composition.

Such changes may represent a transition from a collagen-dominated stromal matrix to an environment more conducive to mineralized tissue formation. Bone morphogenetic proteins (BMPs) are well-known regulators of osteogenic and mineralized tissue differentiation, promoting transcriptional programs linked to mineralized ECM deposition. BMP signaling has been shown to induce the expression of genes related to mineralization, which participate in mineralized matrix formation and ECM remodeling [51]. In line with these mechanisms, our study found increased expression of *SPP1* in experimental SCAP cultures, potentially associated with transcriptional programs related to mineralized matrix deposition.

### Matrix metalloproteinase induction reflecting stress-associated ECM remodeling

In this study, prolonged culture of experimental SCAPs under limited medium renewal conditions was associated with increased expression of *MMP2*, *MMP3*, and *MMP9*. MMPs are zinc-dependent endopeptidases that play vital roles in ECM remodeling [52]. Under pathological conditions, MMP activity is linked to various stages of malignant progression, including tumor growth, angiogenesis, invasion of the basement membrane and stroma, and metastatic dissemination [52]. MMPs are also involved in epithelial–mesenchymal plasticity. Specifically, MMP3 has been shown to induce EMT-related changes, while MMP2 expression is associated with EMT programs in epithelial cells. A bidirectional relationship exists between MMP activity and EMT, where MMPs promote EMT and EMT-associated states further enhance MMP expression [53].

However, outside of oncogenic contexts, elevated MMP expression does not necessarily indicate activation of a canonical EMT program. Rather, the increased expression of *MMP2*, *MMP3*, and *MMP9* observed in this study may reflect an adaptive response involving ECM remodeling under prolonged culture stress. Such stress-induced MMP activity has been reported across various cellular systems, where matrix remodeling contributes to tissue adaptation to environmental or metabolic stress conditions [54].

Notably, we observed increased expression of IL1RN and fibroblast growth factor-binding protein 1 (FGFBP1), further supporting a stress-associated adaptive response. IL1RN encodes the interleukin-1 receptor antagonist, which acts as a natural inhibitor of IL-1–mediated inflammatory signaling, potentially reflecting the activation of anti-inflammatory regulatory mechanisms in experimental SCAPs [55].

The balance between IL-1 and IL-1Ra in disease contexts, along with FGFBP1, which modulates fibroblast growth factor (FGF) signaling by enhancing ligand availability—has been linked to tissue remodeling and implicated in tumor growth as an angiogenic switch molecule [56].

Reactome pathway analysis highlighted the enrichment of cytokine-related pathways, including interleukin signaling; however, this likely reflects the representation of downstream signaling components and pathway crosstalk rather than direct expression of interleukin ligands.

### Activation of odontogenic and enamel-associated gene programs

In SCAPs, the manipulation of BMP2 has been shown to enhance odontogenic differentiation capacity *in vitro* [5]. The increased expression of *SPP1* (osteopontin), a multifunctional glycoprotein and cytokine that regulates ECM remodeling and participates in mineralized matrix interactions under both physiological and pathological conditions, aligns with the activation of matrix-associated and potentially mineralization-linked transcriptional programs [57, 58]**.**

The apical papilla derives from the reduced enamel epithelium, which is responsible for enamel formation and maturation and induces the differentiation of root odontoblasts. SCAPs exhibit multilineage differentiation potential, including odontogenic differentiation, thereby supporting the regeneration of the dentin–pulp complex [59–61]. Notably, the expression of enamel matrix proteins such as amelogenin and ameloblastin in experimental SCAP cells may indicate partial activation of ameloblast-associated pathways and differentiation towards an ameloblast-like phenotype. These proteins are typically secreted by ameloblasts during amelogenesis and serve as key markers for enamel crystal formation and mineralization [62]. Their expression may suggest the engagement of ameloblast-related molecular programs and reflect aspects of epithelial–mesenchymal signaling, with high-density culture conditions potentially mimicking the signaling environment associated with enamel formation. However, as SCAPs are mesenchymal stem cells of dental origin while ameloblasts originate from dental epithelium, the observed expression of enamel matrix proteins alone does not confirm complete ameloblast differentiation. These findings may represent partial activation of ameloblast-related pathways rather than the formation of functional ameloblasts, necessitating further research.

Transcriptional alterations and protein expression detected by fluorescence microscopy do not necessarily indicate terminal differentiation. These observations underscore the need for follow-up assays, such as evaluations of mineralization capacity and odontogenic markers, to assess functional commitment in future studies.

### Limitations

Transcriptional profiling was conducted using a targeted EMT RT^2^ Profiler PCR Array, which examines a defined set of genes and does not capture the full range of transcriptional changes that may occur during long-term high-density culturing. Broader methods, such as RNA sequencing, would provide a more comprehensive view. The expression of enamel-associated proteins detected by fluorescence microscopy was assessed qualitatively and requires further quantitative and functional validation.

RNA samples were pooled prior to RT Profiler PCR Array analysis, preventing gene-level statistical testing and limiting the assessment of variability between individual samples. Pooling was performed to ensure sufficient RNA yield and to capture an overall expression profile across samples, rather than focusing on sample-specific differences. Importantly, the phenotypic changes described in this study were consistently observed across biological replicates prior to pooling for gene expression analysis, and the transcriptional patterns identified were consistent with these observations.

The *in vitro* culture system cannot fully replicate the complexity of the native dental stem cell niche; therefore, the observed transcriptional and phenotypic changes may partly reflect adaptation to artificial culture conditions rather than *in vivo* processes. Additionally, the causal mechanisms underlying these changes were not experimentally tested, and the proposed signaling interactions remain interpretive.

Although increased expression of matrix-remodeling genes (*MMP2*, *MMP3*, *MMP9*) was observed, the study was not designed to specifically investigate these pathways. Thus, their contribution to the observed changes remains indirect.

Despite these limitations, the results provide insight into the coordinated transcriptional and phenotypic changes observed in SCAPs under prolonged high-density culture conditions and highlight the influence of microenvironmental factors during *in vitro* expansion.

## Conclusion

This study provides an exploratory analysis of gene expression changes in SCAPs under prolonged high-density culture conditions with limited medium renewal. While the findings are descriptive, they offer insight into coordinated transcriptional patterns associated with altered cellular phenotypes that have not been previously described in SCAP cultures.

The observed gene expression pattern (↑ *CDH1*, ↑ *KRT14*, ↓ *KRT19*, ↓ *ITGB1/ITGA5*, ↓ *FN1*, ↓ *COL1A2*, ↓ *VCAN*, ↓ *SPARC*, ↓ *SNAI1/SNAI3*, ↓ *ZEB1*, ↑ *MMP2*, ↑ *MMP3*) is consistent with features of a shift toward a more epithelial-like state, characterized by reduced mesenchymal signaling and retained matrix-remodeling capacity.

Increased CDH1 (E-cadherin) expression may promote adherens junction formation and epithelial stabilization, while concurrent decreased expression of *SNAI1*and *ZEB1*, key EMT-inducing transcription factors, is consistent with the attenuation of EMT-associated signaling. Reduced integrin signaling may limit mechanotransduction and ECM synthesis, potentially contributing to increased cell–cell adhesion and reduced migratory potential in prolonged high-density culture conditions. Although TGF-β signaling components were upregulated, their functional activation may be limited by reduced integrin-mediated activation, while concurrent upregulation of BMP7 may counterbalance EMT-promoting signals.

Despite the observed phenotypic and transcriptional changes, experimental SCAP cultures retain expression of genes associated with ECM remodeling and cell–matrix interactions, including *MMP2*, *MMP3*, *AHNAK*, and *SPP1*. This suggests that, even under conditions favoring epithelial stabilization, SCAPs maintain a degree of matrix-remodeling capacity. The coexistence of epithelial stabilization with residual remodeling capacity implies a hybrid cellular state rather than complete lineage transition. Additionally, activation of odontogenic and enamel-associated markers, including enamel matrix proteins, may reflect partial engagement of differentiation-related pathways without evidence of terminal differentiation.

Overall, these findings suggest that prolonged high-density culture under restricted medium renewal is associated with coordinated transcriptional and phenotypic changes in SCAP populations, characterized by reduced expression of canonical EMT regulators, remodeling of ECM programs, and stabilization of epithelial adhesion pathways. The observed changes are consistent with features of an epithelial-like shift, potentially reflecting aspects of mesenchymal-epithelial plasticity and highlighting the sensitivity of dental stem cells to microenvironmental conditions during *ex vivo* expansion.

Further studies using independent biological replicates and functional assays will be necessary to validate these findings and clarify the underlying mechanisms.

## Supplemental data

**Supplemental data 1.** **RT^2^ Profiler PCR Array Gene Expression Analysis Report.** The complete gene expression analysis report generated using the Human EMT RT^2^ Profiler PCR Array and analyzed through the QIAGEN GeneGlobe Data Analysis Center is available in Zenodo: doi:10.5281/zenodo.19187007.

**Supplemental data 2. Reactome Pathway Enrichment Analysis.** The complete Reactome pathway enrichment analysis, including enriched pathways and associated statistical outputs, is available in Zenodo: doi:10.5281/zenodo.19187241.

## Data Availability

All data is provided in text and appendix files. It can be sent to readers upon request.
